# DB-DCAFN: dual-branch deformable cross-attention fusion network for bacterial segmentation

**DOI:** 10.1186/s42492-023-00141-8

**Published:** 2023-07-04

**Authors:** Jingkun Wang, Xinyu Ma, Long Cao, Yilin Leng, Zeyi Li, Zihan Cheng, Yuzhu Cao, Xiaoping Huang, Jian Zheng

**Affiliations:** 1 School of Biomedical Engineering (Suzhou), Division of Life Sciences and Medicine, University of Science and Technology of China, Hefei, 230026 China; 2 Suzhou Institute of Biomedical Engineering and Technology, Chinese Academy of Sciences, Suzhou, 215163 China; 3 Department of Infectious Diseases, the First Affiliated Hospital of Soochow University, Suzhou, 215006 China; 4 Institute of Biomedical Engineering, School of Communication and Information Engineering, Shanghai University, Shanghai, 200444 China; 5 College of Computer and Information, Hohai University, Nanjing, 210098 China; 6 School of Electronic and Information Engineering, Changchun University of Science and Technology, Changchun, 130022 China; 7 Jinan Guoke Medical Technology Development Co., Ltd, Jinan, 250101 China

**Keywords:** Bacterial segmentation, Dual-branch parallel encoder, Deformable cross-attention module, Feature assignment fusion module

## Abstract

Sputum smear tests are critical for the diagnosis of respiratory diseases. Automatic segmentation of bacteria from sputum smear images is important for improving diagnostic efficiency. However, this remains a challenging task owing to the high interclass similarity among different categories of bacteria and the low contrast of the bacterial edges. To explore more levels of global pattern features to promote the distinguishing ability of bacterial categories and maintain sufficient local fine-grained features to ensure accurate localization of ambiguous bacteria simultaneously, we propose a novel dual-branch deformable cross-attention fusion network (DB-DCAFN) for accurate bacterial segmentation. Specifically, we first designed a dual-branch encoder consisting of multiple convolution and transformer blocks in parallel to simultaneously extract multilevel local and global features. We then designed a sparse and deformable cross-attention module to capture the semantic dependencies between local and global features, which can bridge the semantic gap and fuse features effectively. Furthermore, we designed a feature assignment fusion module to enhance meaningful features using an adaptive feature weighting strategy to obtain more accurate segmentation. We conducted extensive experiments to evaluate the effectiveness of DB-DCAFN on a clinical dataset comprising three bacterial categories: *Acinetobacter baumannii*, *Klebsiella pneumoniae*, and *Pseudomonas aeruginosa*. The experimental results demonstrate that the proposed DB-DCAFN outperforms other state-of-the-art methods and is effective at segmenting bacteria from sputum smear images.

## Introduction

Lower respiratory tract infections are a significant public health issue because of the ease of person-to-person transmission, resulting in an even greater burden than cancer or heart disease [1, 2]. *Acinetobacter baumannii* (Aba) [3], *Klebsiella pneumoniae* (Kpn) [4], and *Pseudomonas aeruginosa* (Pae) [5] are three common gram-negative bacteria that have been confirmed to cause respiratory tract infections. The sizes of Aba, Kpn, and Pae are generally in the range of (0.6-1.0) um × (1.0-1.6) um, (0.5-0.8) um × (1-2) um, and (0.5-1) um × (1.5-5) um, respectively. Aba and Kpn have no endospores and flagella, whereas Pae has no endospores and has only one flagellum at one end. Under the microscope, Aba, Kpn, and Pae are spherical or club-shaped, rod-shaped, and rod-shaped or linear, respectively. Generally, Aba is arranged individually or in pairs; Kpn is arranged individually, in pairs, or in short chains; and Pae is arranged in pairs or short chains. The sputum smear test [6] and sputum culture test [7] are two common tools used to identify bacteria in sputum. The sputum culture test has a high sensitivity; however, it requires the physician to inoculate a portion of the sputum sample onto a petri dish to cultivate a large number of target bacteria and then identify the bacterial category. This process is cumbersome and time consuming. The sputum smear test requires only a physician to observe the stained bacteria under a microscope, and has the advantages of rapid diagnosis, simplicity of operation, and low cost. However, the size ranges of the club-shaped Aba, rod-shaped Kpn, and Pae highly overlap, and their morphological features under the microscope are relatively similar, which can easily lead to misjudgment. In addition, manually calculating clinical information, such as the bacterial area ratio, is a tedious and time-consuming task. Therefore, it is important to develop a computer-aided diagnostic system that can accurately segment and identify different bacterial categories in sputum smear images.

Morphology-based methods have been used in sputum smear tests. Makkapati et al. [8] proposed a hue color component-based method to segment bacilli in Ziehl-Neelsen (ZN)-stained sputum smear images. This method adaptively selects the hue range to segment bacilli and then removes invalid bacilli and other artifacts by thresholding the area, thread length, and thread width. Khutlang et al. [9] used a combination of two-class pixel classifiers to segment candidate Bacillus objects from ZN-stained sputum smear images and extracted geometric-transformation-invariant features to identify *Mycobacterium tuberculosis*. Sadaphal et al. [10] proposed a multistage color-based Bayesian segmentation method to identify *Mycobacterium tuberculosis* in sputum smear images. Priya and Srinivasan [11] used an active contour method to segment candidate *Mycobacterium tuberculosis* objects from sputum smears, including bacilli and outliers. After obtaining the initial segmentation result, they used 15 Fourier descriptors (FDs) to describe the boundary of the segmented objects and fed the FDs as input to support vector neural networks to distinguish between bacilli and outliers. However, these methods have the following limitations: (1) a large number of manual parameters need to be set and adjusted carefully; (2) the entire process is cumbersome; and (3) only one category of bacteria is segmented and identified, which needs to be verified when generalized to multi-class bacteria segmentation tasks.

**Fig. 1:**
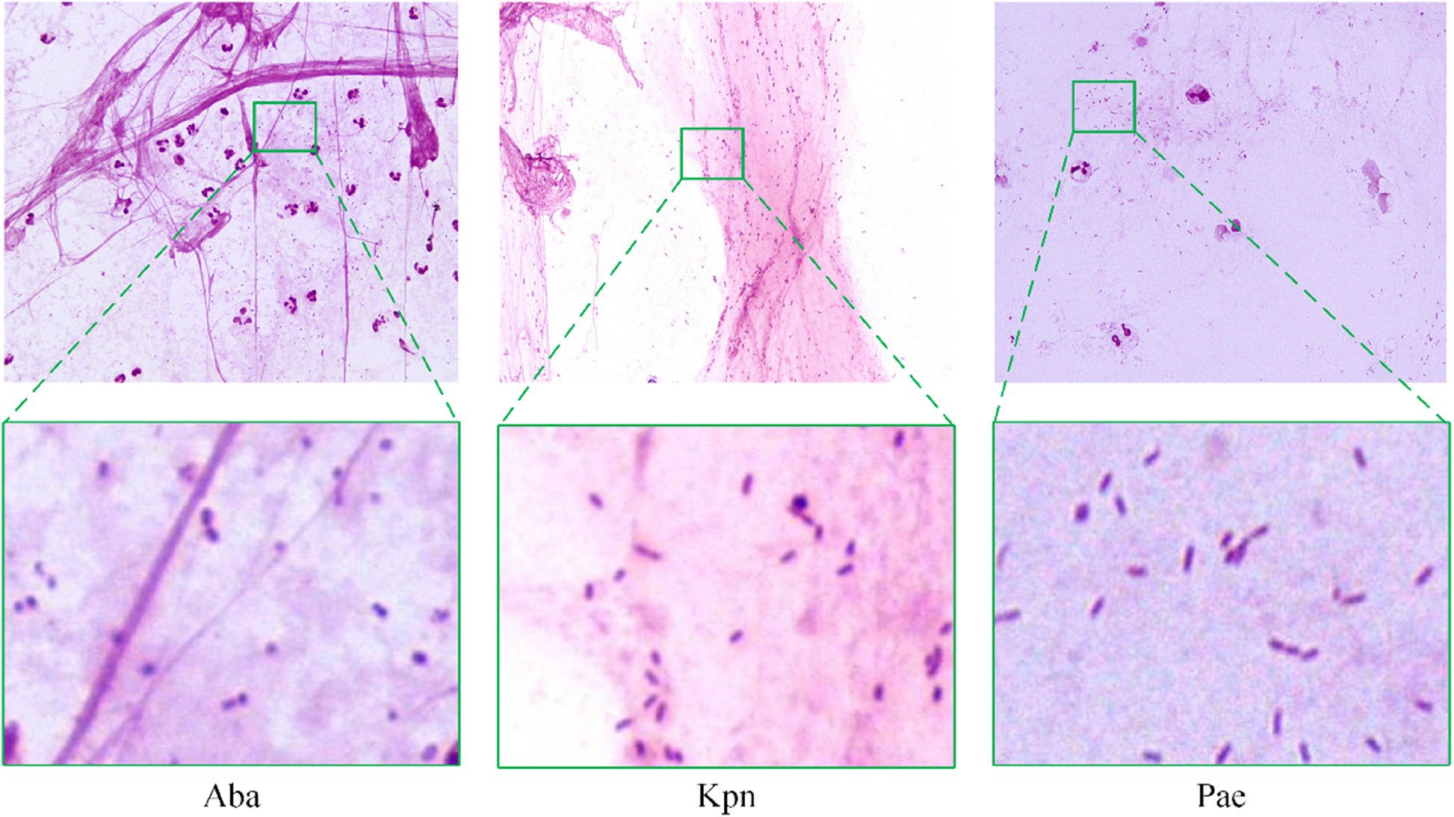
Examples of the sputum smear image. The bacteria in the green box are enlarged to show their morphology. Bacterial categories including Aba, Kpn, and Pae

In recent years, convolutional neural network (CNN)-based methods have become popular in semantic segmentation, including the famous fully convolutional network (FCN) [12]. Furthermore, the U-Net proposed by Ronneberger et al. [13] adds the “skip connection” to FCN to alleviate the loss of shallow information, which has demonstrated excellent performance on many segmentation tasks, and U-Net-based methods have been applied to many segmentation tasks [14–16]. Angayarkanni et al. [17] enhanced the quality of sputum smears using several image preprocessing methods and then fed the preprocessed images to U-Net for *Mycobacterium tuberculosis* segmentation. Ali et al. [18] proposed a modified U-Net and applied it to the segmentation of bacilli in ZN-stained sputum smear images. Reddy et al. [19] used ResU-Net + + to segment and identify different categories of bacteria, such as Aba and *Escherichia coli* (Eco), in gram-stained sputum smear images. Although U-Net-based methods can distinguish bacteria from the background, bacterial categories are frequently misclassified. This may be because the morphologies of different categories of bacteria are very similar, as shown in Fig. 1, and the limited features extracted by local convolution are insufficient to distinguish between them. To improve the inherent deficiency of the insufficient global information extraction ability of the convolution operation, some researchers [20–22] have applied the self-attention mechanism [23] with long-range dependency modeling ability to CNNs with impressive results. Furthermore, the Vision Transformer [24], which consists of transformers with global self-attention, has achieved SOTA results on ImageNet classification. To achieve better segmentation performance, many studies [25–27] have applied convolution together with a transformer to the encoder of the network to extract local features and global context information simultaneously. However, these methods only fuse the information extracted by the CNN and transformer by simply adding or concatenating them, ignoring the semantic gap caused by different modeling patterns. This issue hinders further improvements in segmentation performance. Therefore, integration of local features pertaining to bacterial morphology and global context features pertaining to bacterial distribution remains a challenge.

In this paper, we propose a novel dual-branch deformable cross-attention fusion network (DB-DCAFN) for accurate bacteria segmentation. Specifically, we designed a dual-branch parallel encoder to simultaneously extract multilevel local and global features to ensure accurate bacterial localization and category judgement. We further designed a deformable cross-attention (DCA) module that models the potential relationship between local and global features, performs deformable feature sampling, and selects sparse and effective features for cross-attention, thus bridging the semantic gap during feature fusion to further improve the segmentation performance. Additionally, we designed a feature assignment fusion (FAF) module to enhance meaningful features during skip connections, which improves the fusion efficiency of the encoded and decoded features. Extensive experiments demonstrate that the proposed DB-DCAFN outperforms other state-of-the-art (SOTA) methods for bacterial segmentation. The main contributions of this study are as follows:

1. A novel multistage CNN and transformer parallel network, DB-DCAFN, was proposed for accurate bacterial segmentation. Unlike traditional CNN-based, transformer-based, or CNN-transformer cascaded encoders, the encoder of the DB-DCAFN adopts a dual-branch form, including multiple parallel ResNet blocks and Swin Transformer blocks, which can adequately capture multilevel local and global features.
2. A novel DCA module is proposed to bridge the semantic gap during feature fusion. The DCA module can capture the semantic dependencies between different features using the cross-attention mechanism and reduce the computational cost by deformable feature sampling, further improving the performance of bacterial segmentation. In addition, a novel FAF module was proposed to adaptively enhance meaningful features during skip connections, ensuring that the decoder could efficiently integrate features to obtain accurate segmentation results.
3. The effectiveness of DB-DCAFN was evaluated using a clinical sputum smear dataset. Comparative experiments with other SOTA segmentation networks demonstrate the superior performance of the DB-DCAFN. Detailed ablation experiments further demonstrate the validity of the proposed modules.

## Methods

### Overview

**Fig. 2:**
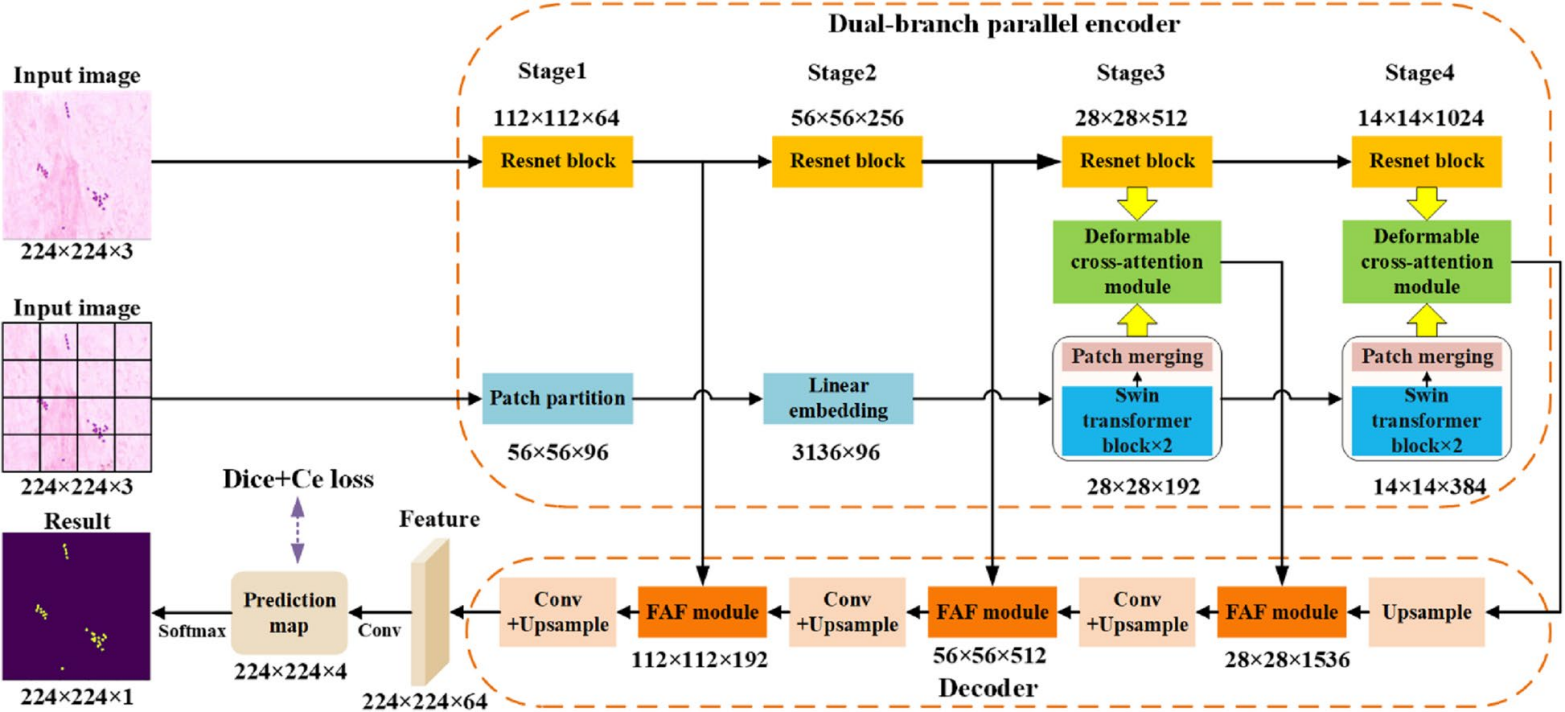
Overview of the proposed DB-DCAFN. The purple dotted line represents the supervision

Figure 2 presents an overview of the proposed DB-DCAFN, which comprises a dual-branch parallel encoder with a DCA module and a decoder with a FAF module. The output of the decoder is a feature map of size 224 × 224 × 64, which is used to generate a prediction map through 3 × 3 and 1 × 1 convolutional layers. There are four channels in the prediction map, corresponding to the probability of each pixel being predicted as Aba, Kpn, Pae, and background. Finally, a softmax function was applied to the channel of each pixel in the prediction map to obtain a specific category corresponding to each pixel. The dual-branch parallel encoder consists of multiple ResNet [28] and Swin Transformer [29] blocks, which are designed to fully capture both local and global context features to facilitate accurate bacterial localization and classification. The DCA module bridges the semantic gap between different features, thereby achieving more efficient feature fusion to promote feature utilization. In addition, the FAF module is used to enhance meaningful features during the skip connection by using an adaptive feature weighting strategy. The proposed components are detailed in the following section.

### Dual-branch parallel encoder

To accurately distinguish between target and background pixels and recognize the categories of bacteria simultaneously, the segmentation network must not only be able to extract rich local features but also be effectively supplemented with global pattern information. To this end, we designed a dual-branch parallel encoder comprising a CNN branch and transformer branch, as shown in Fig. 2. Specifically, the CNN branch utilizes ResNet-50 to extract multilevel local features. The four ResNet blocks in Fig. 2 were obtained from the first four stages of ResNet-50, and each block extracted the features of the input by convolution. Although CNN encoder-based methods have achieved tremendous success in segmentation tasks, the global pattern information and long-range dependencies extracted by simply repeating the convolution are limited. Inspired by the unique global context dependency modeling pattern in the transformer, we employ the Swin Transformer as the other encoder branch to capture global context information. By introducing a shift window in the multi-head self-attention mechanism, the Swin Transformer improves the performance while significantly reducing the computation cost. Specifically, a Swin Transformer block consists mainly of a window-based multi-head self-attention (W-MSA) module and a shifted window-based MSA module (SW-MSA). The W-MSA module can directly model the dependencies between different features within a window, whereas the window partition cyclic shift in the SW-MSA module enables information interaction between adjacent windows, further ensuring the extraction of contextual features. Compared to the ResNet block at the same stage, the Swin Transformer is better at extracting global context information, such as long-term dependency. In contrast to other two-branch feature extraction strategies [25] that only capture global features at a single semantic level, the proposed two-branch parallel encoder can extract multilevel global features, thus providing more information to facilitate correct predictions.

### DCA module

**Fig. 3:**
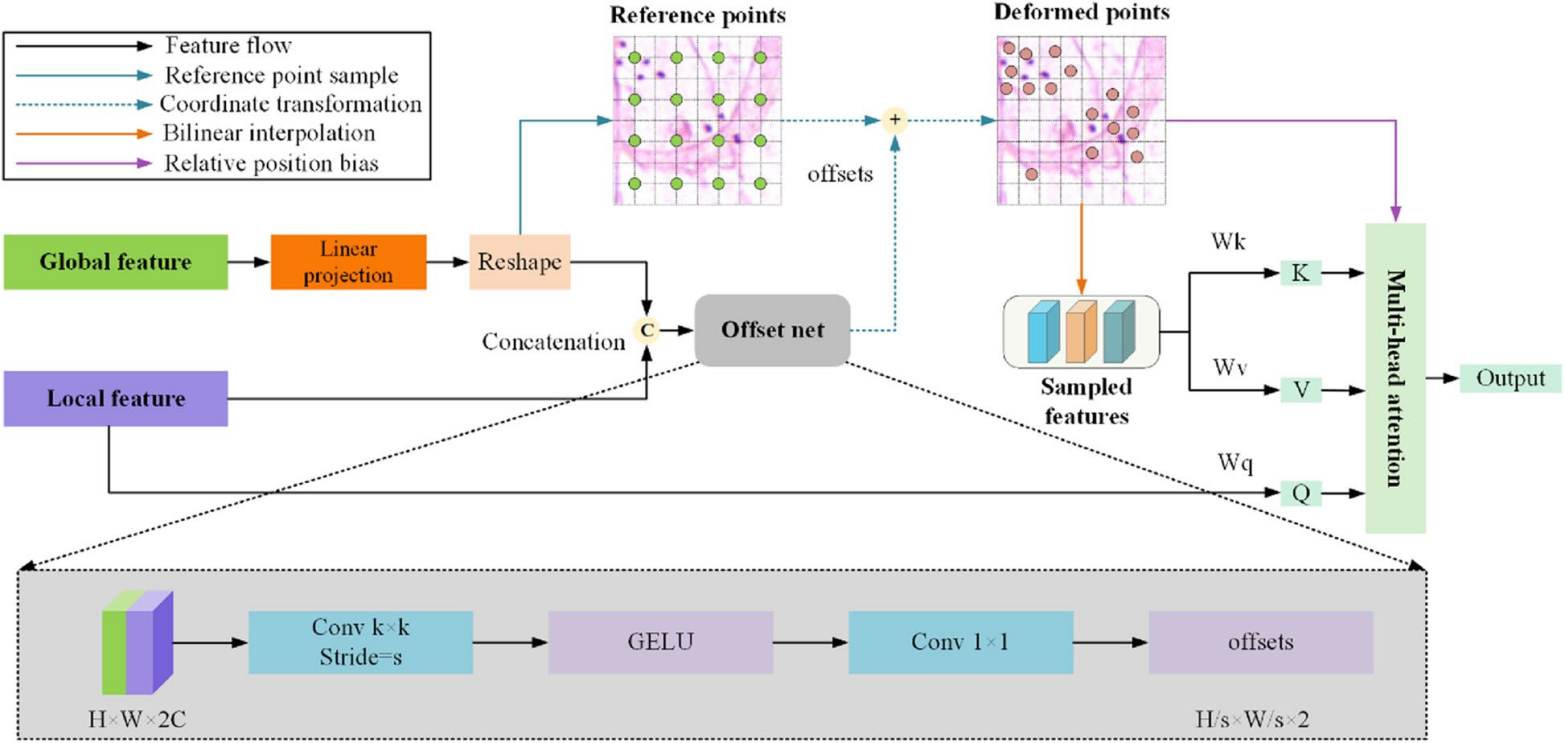
DCA module

After obtaining the local and global features from the encoder, it is crucial to integrate these two semantically distinct features effectively. A direct cascade or summation is a common method for fusing these two features [25, 26]. However, there is a semantic gap between them owing to the difference in the modeling patterns of the CNN and transformer [30]. In addition, local and global features are misaligned in space [31]. Therefore, a rough and predefined integration strategy limits segmentation performance. Cross-attention [32] has recently been shown to have significant potential for capturing semantic dependencies between different features and bridging the semantic gap during feature fusion. However, the high computational cost of cross-attention hinders its application in the multiple stages of an encoder. The deformable attention (DAT) module [33] shares sampled keys and values for each query in the feature map for an efficient computational tradeoff. Inspired by this, we propose a DCA module that can adaptively assign sparse but more valuable keys and values in the global feature map to each query in the local feature map for multi-head cross-attention. Figure 3 shows the specific process of the DCA module, which takes two inputs: the global features captured by the Swin Transformer block and the local features captured by the ResNet block. The global features are first passed through a linear layer so that their channel dimensions are consistent with the local features and then restored to the original $\mathrm{B}\times \mathrm{H}\times \mathrm{W}\times \mathrm{C}$ dimensions. The DCA module exploits the potential correlations between local and global features through a lightweight offset network and generates deformable sampling points accordingly. The features on these deformable sampling points represent potentially valuable global features, which are then fed into multi-head cross-attention along with local features for sparse and effective feature fusion. This process can be divided into the following two steps:

(1) **Deformable points generation:** Similar to DAT, we used the offset network to add an offset to the reference coordinate points to generate deformable points. Specifically, the reference points $P\in {\mathbb{R}}^{{H}_{p}\times {W}_{p}\times 2}$ are obtained by sampling the coordinate points of the current feature map $F\in {\mathbb{R}}^{H\times W\times C}$ with a sampling rate of $S$, so that ${H}_{p}=H/S$, ${W}_{p}=\mathrm{W}/S$. The values of the reference points are linearly spaced 2D coordinates $\left\{\left(\mathrm{0,0}\right),\dots ,({H}_{p}-1,{W}_{p}-1)\right\}$, and we normalized them to the range [-1, 1]. We then cascaded the global and local features and fed them into the offset network to generate the offset $O\in {\mathbb{R}}^{{H}_{p}\times {W}_{p}\times 2}$. Offset $O$ is added to reference point $P$ to obtain the deformable point coordinates, which can be formulated as follows:

1 $${P}_{(x,y)}^{*}={P}_{(x,y)}+{O}_{(x,y)}$$

where ${P}_{(x,y)}^{*}$ represents the deformable point.

The offset network consists of convolution layers, layer normalization, and GELU activation. The first convolution has a kernel size of $k$, a padding value of $k//2$, and a stride value of $S$, which ensures that the spatial resolution of offset *O* is ${H}_{p}\times {W}_{p}$. The last convolution has a kernel size of 1 and an output channel of 2, ensuring that the number of channels for offset $O$ is the same as the dimension of the reference point $P$. The specific offset network process is formulated as follows:

2 $$offset={conv}_{2}(GELU(LN\left({conv}_{1}\left({F}_{c},{w}_{1}\right)\right)),{w}_{2})$$

where ${conv}_{1}$ and ${conv}_{2}$ represent the first and last convolutional layers, respectively, and ${w}_{1}$,${w}_{2}$ represent their respective parameters. $LN$ refers to layer normalization, *GELU* refers to GELU activation, ${F}_{c}\in {\mathbb{R}}^{H\times W\times 2C}$ represents the input feature cascaded by local and global features, and $offset\in {\mathbb{R}}^{{H}_{p}\times {W}_{p}\times 2}$ represents the output of the offset network. In our experiments, stride $S$ was set to 2.

(2) **Deformable multi-head cross-attention:** After obtaining the deformable sample points, sparse but more valuable global features can be sampled on the global feature map accordingly. To make this sampling process differentiable, the global features were sampled using a bilinear interpolation method as follows:

3 $${F}_{g({p}_{x}^{*},{p}_{y}^{*})}^{*}=\sum_{({p}_{x},{p}_{y})}g\left({p}_{x}^{*},{p}_{x}\right)g\left({p}_{y}^{*},{p}_{y}\right){F}_{g}[{p}_{x},{p}_{y},:]$$

where ${F}_{g}$ $\in {\mathbb{R}}^{H\times W\times C}$ represents the original global features, ${F}_{g({p}_{x}^{*},{p}_{y}^{*})}^{*}$ represents the sampled global features at $({p}_{x}^{*},{p}_{y}^{*})$, $({p}_{x},{p}_{y})$ indexes all the locations on ${F}_{g}$, $({p}_{x}^{*},{p}_{y}^{*})$ represents the coordinates of the deformation point ${P}_{(x,y)}^{*}$,and $g\left(a,b\right)=\mathrm{max}(\mathrm{0,1}-\left|a-b\right|)$. The formulation of $g$ ensures that ${F}_{g({p}_{x}^{*},{p}_{y}^{*})}^{*}$ is obtained by the weighted addition of the features on the four reference points closest to the deformation point ${P}_{(x,y)}^{*}$. Next, we multiply the local features ${F}_{l}$ by the ${W}_{q}$ projection matrix to obtain the query embeddings $q$, and multiply the sampled global features ${F}_{g}^{*}$ by ${W}_{k}$ and ${W}_{v}$ to obtain the deformed key embeddings ${k}^{*}$ and value embeddings ${v}^{*}$, respectively, as follows:

4 $$q={F}_{l}{W}_{q}, {k}^{*}={F}_{g}^{*}{W}_{k}, {v}^{*}={F}_{g}^{*}{W}_{v}$$

Finally, we perform multi-head cross-attention on the obtained $q$, ${k}^{*}$, and ${v}^{*}$, as follows:

5 $${F}_{out}=SoftMax(\frac{q{{k}^{*}}^{T}}{\sqrt{d}}+\widetilde{B}){v}^{*}$$

where $\widetilde{B}$ represents the relative position embedding and ${F}_{out}$ represents the multi-head cross-attention results.

In contrast to the original cross-attention mechanism, the DCA module can model the potential relationship between local and global features and sample more meaningful global features, effectively bridging the semantic gap and significantly reducing the computational cost. It can be used in multiple stages of the encoder to integrate features at different levels, facilitating the identification of bacteria and correctly classifying pixels inside the bacteria.

### FAF module

**Fig. 4:**
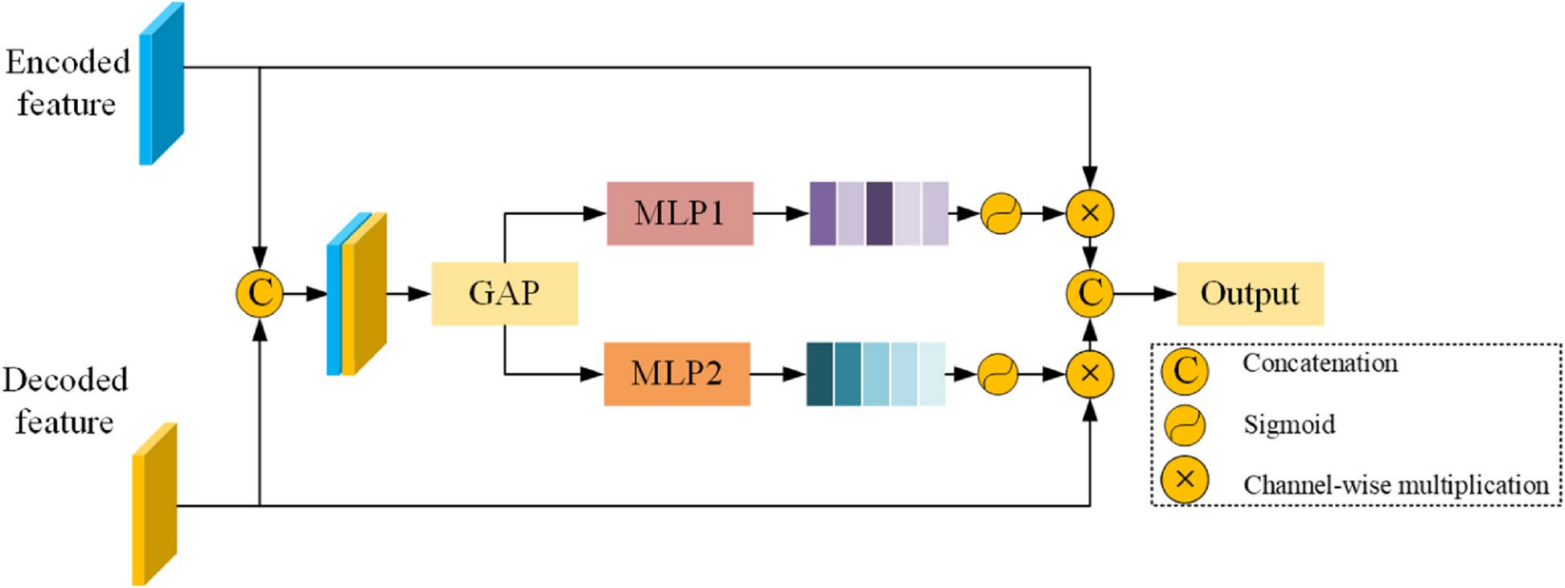
FAF module

The introduction of spatial fine-grained features at the decoding stage is essential for the network to correctly segment small bacteria. Although the skip connection can help recover detailed spatial information that is lost owing to pooling operations, directly cascading low-level encoded features with high-level decoded features may result in redundant useless information. To address this problem, we propose a FAF module that can adaptively enhance meaningful features during skip connections. The details of the proposed FAF block are shown in Fig. 4. We first cascade the encoded and decoded features, and then use a global average pooling operation to squeeze the spatial information of the cascaded features, which can be formulated as follows:

6 $${F}_{fuse}=GAP(concat({F}_{encoded},{F}_{decoded}))$$

where ${F}_{encoded}$ and ${F}_{decoded}$ represent the encoded and decoded features, respectively, $GAP$ and $concat$ represent the global average pooling operation and concatenation, respectively, and ${F}_{fuse}$ represents the cascaded features.

After obtaining the cascaded features, they were passed through two $MLP$ layers to obtain two independent channel weight maps. We then use these two weight maps to enhance the encoded and decoded features and re-cascade the enhanced features to generate the final output features, which can be expressed as follows:

7 $${F}_{encoded}^{*}={E}_{scale}({MLP}_{1}\left({F}_{fuse}\right),{F}_{encoded})$$

8 $${F}_{decoded}^{*}={E}_{scale}({MLP}_{2}\left({F}_{fuse}\right),{F}_{decoded})$$

9 $${F}_{out}=concat({F}_{encoded}^{*},{F}_{decoded}^{*})$$

where ${E}_{scale}$ represents channel-wise multiplication, ${F}_{encoded}^{*}$ and ${F}_{decoded}^{*}$ represent the enhanced encoded and decoded features, respectively, and ${F}_{out}$ represents the output of the FAF module.

In the proposed network, the FAF module is used in three skip connection processes to promote the utilization of low-level encoded and high-level decoded features, ensuring that the model can accurately segment small bacteria.

### Loss function

As shown in Fig. 2, we used a joint loss consisting of the cross-entropy and Dice losses to optimize the DB-DCAFN. The loss function is formulated as follows:

10 $$Loss= {Loss}_{ce}+{Loss}_{dice}$$

11 $${Loss}_{ce}\left(y,p\right)=-\frac{1}{N}\sum_{i=1}^{N}\sum_{c=1}^{M}{y}_{ic}log({p}_{ic})$$

12 $${Loss}_{dice}\left(y,p\right)=1-\frac{2\sum_{i=1}^{N}\sum_{c=1}^{M}{y}_{ic}{p}_{ic}}{\sum_{i=1}^{N}\sum_{c=1}^{M}({y}_{ic}+{p}_{ic})}$$

where $y$ is the ground truth, $p$ is the network prediction, $N$ is the number of pixels, and M is the number of classes. ${\gamma }_{ic}$ is an indicator function that equals 1 if the class of $i-th$pixel is $c$ and 0 otherwise.${p}_{ic}$ denotes the probability that the $i-th$pixel is predicted to be of class $c$.

## Results

### Dataset

We conducted detailed experiments on a clinical gram-stained sputum smear dataset provided by the Department of Infectious Diseases at The First Hospital of Soochow University. All sputum smears were collected by electron microscopy at 100 × magnification, and the bacteria in the sputum smears were labeled by physicians using semi-automatic labeling software. The dataset contained 273 sputum smear images from 30 patients, and each image had a resolution of 2448 × 2048 pixels. Examples of the sputum smear images are shown in Fig. 1. This dataset contained three common bacteria: Aba, Pae, and Kpn, with 10 cases for each bacterial category. To make the experiment convincing, the entire dataset was randomly split into training, validation, and test sets at the case level; five cases from each bacterial category were selected as the training set, three cases as the test set, and two cases as the validation set. The number of sputum smear images of different bacterial categories in the three sets is shown in Table 1.

**Table 1:** Details of the Gram-stained sputum smear dataset division

	Aba	Kpn	Pae	All
Training set	48	44	36	128
Validation set	20	19	20	59
Test set	27	30	29	86
All	95	93	85	273

### Implementation details

The proposed segmentation network is implemented using PyTorch on an NVIDIA GeForce RTX 3060 GPU card. To reduce computational cost and make full use of the data, we randomly cropped 224 × 224 image patches from the original 2448 × 2048 sputum smear image and fed them into the network during the training process. For all sputum smear images in the validation and test sets, we used a sliding window of size 224 × 224 to perform local segmentation, and combined the results of all windows. We also applied three types of online data augmentation, namely, image scaling, horizontal flipping, and hue adjustment, to avoid overfitting during training. We did not use any pretrained weights to train the proposed DB-DCAFN. Stochastic gradient descent was adopted as the optimizer with the weight decay and momentum set to 0.0001 and 0.9, respectively. The initial learning rate was set to 0.01, and the batch size was set to 12.

For compatibility purposes, the final pooling and fully connected layers of ResNet-50 were removed, and the 7 × 7 convolutional layers of the first stage in ResNet-50 were replaced with three 3 × 3 convolutional layers to maintain more detailed information. The second, third, and fourth stages of ResNet-50 are composed of three, six, and nine bottleneck blocks, respectively, and the dimensions of the output features of these four stages are 64, 256, 512, and 1024, respectively. Moreover, group normalization was employed to replace batch normalization in ResNet-50 to increase the robustness of the model. In the Swin Transformer layer, the patch size was set to four, and the window size was set to seven, the same as in the original Swin Transformer. In the first and second DCA modules, the first convolution kernel size of the offset network was set to 5 × 5 and 3 × 3, the stride was set to 2, and the head numbers in the multi-head cross-attention were set to 16 and 32.

### Evaluation metrics

To evaluate the experimental results quantitatively, we adopted the following two metrics that are widely used in the field of semantic segmentation: the Dice similarity coefficient (Dice) and the Jaccard index. The calculation formulae are as follows:

13 $$Dice= \frac{2TP}{2TP+FP+FN}$$

14 $$Jaccard= \frac{TP}{TP+FP+FN}$$

where $TP$ represents the number of correctly classified target pixels, $FP$ represents the number of incorrectly classified target pixels, and $FN$ represents the number of incorrectly classified background pixels.

### Comparison with other SOTA methods

To validate the performance of the proposed DB-DCAFN, we compared it with five methods that exhibit excellent performance in semantic segmentation: U-Net [13], CE-Net [34], Swin-Unet [35], TransUNet [26], and UCTransNet [36]. U-Net is a classic network used for medical image segmentation, and CE-Net is a modification of U-Net. Swin-Unet replaced the convolution modules in U-Net with Swin Transformer modules and achieved SOTA performance in many medical image-segmentation tasks. TransUNet employs a hybrid CNN-transformer architecture to leverage both detailed high-resolution spatial information and global context information, and UCTransNet proposes a channel-wise cross-fusion transformer to bridge the semantic gap between multi-scale context features. For a fair comparison, the originally released code and published settings for these comparison methods were used in the experiments. In addition, all competitors used the same loss functions, data augmentations, and sliding window sizes as those used in our study.

We calculated the average Dice and Jaccard metrics for each category of bacterial data and all data in the test set. The results are presented in Table 2. Overall, U-Net exhibited the worst segmentation performance, with an average Dice and Jaccard score of 62.35% and 47.76%, respectively, for the entire test set. Benefitting from the dense atrous convolution and residual multi-kernel pooling modules, CE-Net can capture more context information, achieving better segmentation performance than U-Net, with an improvement of 2.06% and 1.93% on Dice and Jaccard, respectively, for the entire test set. UCtransNet uses a channel-wise transformer to fuse the features at different scales, compared with CE-Net, it achieves a Dice improvement of 0.64%. Swin-Unet uses multiple Swin Transformer blocks to form its encoder and applies patch-expanding layers in the decoding stage to perform the up-sampling operation. The overall performance of Swin-Unet was slightly better than that of UCTransNet. TransUNet uses a transformer to extract global context information to enhance representation capabilities and uses a CNN to extract high-resolution local features to enable precise localization. Based on the exploitation of global and local features, it outperformed Swin-Unet, with an increment of 1.68% and 2.06% in terms of Dice and Jaccard, respectively. Our DB-DCAFN achieved the highest scores in almost all metrics: 70.57% and 55.54% on Dice and Jaccard for Aba, 65.36% and 51.55% for Kpn, 70.49% and 56.40% for Pae, and 68.73% and 54.44% for Dice and Jaccard for the entire test set, respectively. These experimental results demonstrate that our method outperforms other SOTA methods and is effective for bacterial segmentation.

**Table 2:** Results of comparison experiments on bacteria segmentation task

Methods	Dice (mean ± SD, %)				Jaccard (mean ± SD, %)			
	Aba	Kpn	Pae	All	Aba	Kpn	Pae	All
U-Net	61.58 ± 14.85	59.26 ± 21.21	66.28 ± 18.08	62.35 ± 18.58	46.09 ± 15.04	45.14 ± 20.29	52.04 ± 18.45	47.76 ± 18.41
CE-Net	67.74 ± 14.86	59.48 ± 21.23	66.41 ± 13.87	64.41 ± 17.46	52.97 ± 15.66	45.35 ± 20.23	51.13 ± 13.71	49.69 ± 17.14
UCtransNet	62.75 ± 15.86	63.35 ± 16.41	68.96 ± 17.60	65.05 ± 16.89	47.58 ± 16.20	48.32 ± 16.44	55.00 ± 17.96	50.34 ± 17.22
Swin-Unet	67.41 ± 9.52	59.15 ± 21.40	69.19 ± 16.79	65.13 ± 17.40	51.57 ± 10.19	45.00 ± 19.92	55.26 ± 18.64	50.52 ± 17.51
TransUNet	**70.64 ± 8.99**	61.80 ± 20.80	68.43 ± 19.52	66.81 ± 17.86	55.35 ± 10.75	47.78 ± 20.45	54.97 ± 20.23	52.58 ± 18.24
Ours	70.57 ± 10.63	**65.36 ± 20.62**	**70.49 ± 14.90**	**68.73 ± 16.26**	**55.54 ± 12.47**	**51.55 ± 19.78**	**56.40 ± 17.24**	**54.44 ± 17.03**

In addition, we investigated the speed of training and inference for different models using the same hardware. The experimental results are listed in Table 3, which shows that the training speed and inference speed of our model are at the middle level among the comparison methods, indicating that the proposed modules do not significantly increase the computational cost.

**Table 3:** Results of comparison experiments on training speed and inference speed for different models

Methods	Training speed (s/epoch)	Inference speed (ms/image)
U-Net	**3.79**	11.87
CE-Net	4.97	26.03
UCtransNet	8.94	32.61
Swin-Unet	4.04	**11.77**
TransUNet	7.55	19.56
Ours	5.93	20.36

### Visual segmentation results of bacteria on sputum smear images

**Fig. 5:**
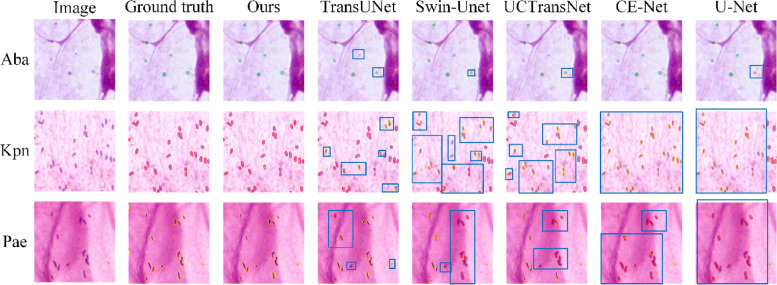
Visualized segmentation results of different methods for bacteria with different categories. From top to bottom, there are image patches containing Aba, Kpn, and Pae. The contours of Aba, Kpn and Pae are depicted by green, red and yellow lines respectively, and misclassified bacteria are highlighted in blue boxes

Different categories of bacteria may have similar morphologies, therefore, pixel recognition errors within bacteria are the main limitation of segmentation performance. In this section, we evaluate the performance of the proposed DB-DCAFN in identifying different bacterial categories. Several visual segmentation results are shown in Fig. 5, where the contours of Aba, Kpn, and Pae in the ground truth and segmentation results are depicted by green, red, and yellow lines, respectively, and some misclassified bacteria are highlighted in blue boxes. U-Net and CE-Net can distinguish bacteria well from the background, but they generate numerous misclassifications within the categories of bacteria: a large number of Kpn and Pae are confused. This phenomenon may be due to the difficulty in distinguishing bacteria with similar morphologies based only on local features extracted by the CNN. Owing to the application of the transformer, both UCTransNet and Swin-Unet improved the recognition of Pae and Kpn but still misclassified some bacteria. Compared to the above methods, TransUNet significantly improves the identification accuracy of the three bacteria, as shown in Fig. 5, indicating that both local and global contextual features are essential for accurate bacterial segmentation. It is worth noting that the proposed DB-DCAFN achieved the best performance in these cases, correctly identifying the majority of bacteria and demonstrating that it can effectively capture both local and global features. The improvement compared to TransUNet proves that our DCA module can effectively bridge the semantic gap between local and global features, further promoting the accurate segmentation of bacteria.

**Fig. 6:**
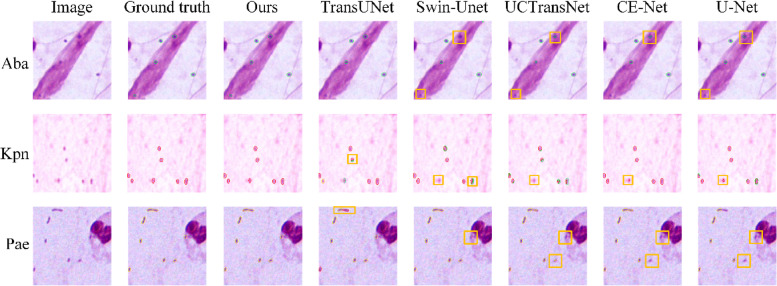
Visualized segmentation results of different methods for ambiguous bacteria. The contours of Aba, Kpn and Pae are depicted by green, red and yellow lines respectively, and some bacteria that are missed or insufficiently segmented are highlighted in orange boxes

In addition to the high interclass similarity among the different bacterial categories, some blurred bacteria may be hidden in the background, thereby increasing the difficulty of segmentation. In this section, we describe the performances of different methods on these ambiguous bacteria. As shown in Fig. 6, the two Aba bacteria hidden in the background region (highlighted in orange boxes) were missed by Swin-Unet. This indicates that it is difficult to locate these small and ambiguous targets by relying solely on global context features. Other comparative methods also have varying degrees of missed detection or insufficient segmentation, which may be due to the masking of useful features in the decoding stage. Similarly, several ambiguous Kpn and Pae (highlighted in orange boxes) were missed or insufficiently segmented using these comparative methods. Notably, the proposed method successfully detected these ambiguous bacteria and the segmentation results were very close to the ground truth. This can be attributed to the FAF module, which adaptively enhances useful features during the fusion of high-resolution encoded and high-semantic decoded features. In addition, sufficient local features extracted by the dual-branch encoder and effective feature aggregation in the DCA module ensured the ability of the model to locate these small and ambiguous bacteria.

### Ablation experiments

In this section, we conducted detailed ablation experiments to demonstrate the effectiveness of each module. Firstly, we used two baseline networks: one with a CNN encoder, and the other with a transformer encoder. The baseline network with the CNN encoder was based on the U-Net architecture, and its encoder was ResNet-50 to ensure sufficient feature extraction and avoid gradient disappearance during training. In addition, the original 5-layer encoder-decoder structure was replaced with a 4-layer structure for better performance and reduced computational cost. The hyperparameters of ResNet-50 were consistent with those in Implementation details section. The baseline network with the transformer encoder is also based on the U-Net architecture, and its encoder consists of two Swin Transformer layers, each of which has two Swin Transformer blocks and a patch-merging block, whereas its bottleneck is also composed of two Swin Transformer blocks. In ablation studies, all methods used the same data augmentation and hardware environment to guarantee a fair comparison. The experiment results are listed in Table 4, and it can be seen that the baseline network with CNN encoder achieves 69.05% on Dice for Aba, 57.07% for Kpn, 66.33% for Pae, and 63.95% for all categories of bacteria, respectively, and the baseline network with the transformer encoder achieves 67.07% on Dice for Aba, 60.09% for Kpn, 65.92% for Pae, and 64.25% for all categories of bacteria.

**Table 4:** Quantitative evaluation of ablation experiments of each change to baseline

Methods	Dice (mean ± SD, %)			
	Aba	Kpn	Pae	All
Baseline1(CNN encoder)	69.05 ± 17.43	57.07 ± 23.56	66.33 ± 16.50	63.95 ± 18.12
Baseline2(transformer encoder)	67.07 ± 10.15	60.09 ± 21.98	65.92 ± 20.63	64.25 ± 18.81
Baseline1 + Dual-branch encoder	68.89 ± 8.73	59.07 ± 21.68	68.89 ± 12.35	65.46 ± 16.16
Baseline1 + Dual-branch encoder + DCA	68.81 ± 11.32	63.73 ± 22.41	69.26 ± 9.67	67.19 ± 15.92
Baseline1 + Dual-branch encoder + DCA + FAF (ours)	**70.57 ± 10.63**	**65.36 ± 20.62**	**70.49 ± 14.90**	**68.73 ± 16.26**

#### (1) Ablation experiments for dual-branch parallel encoder

To verify the effectiveness of the dual-branch encoder, we replaced the original encoder in the baseline network containing the CNN encoder with it, and integrated the features extracted by the ResNet and Swin Transformer blocks through direct concatenation and convolution. Compared to the Baseline with CNN encoder, the Dice of Kpn and Pae improved from 57.07% to 59.07% and from 66.33% to 68.89%, respectively, after applying the dual-branch encoder, although the Dice of Aba decreased slightly. Overall, the Dice for all bacterial categories improved by 1.51%. Compared to the Baseline with transformer encoder, the Dice of Aba and Pae improved by 1.82% and 2.97%, respectively, after applying the dual-branch encoder, and the Dice for all categories of bacteria improved by 1.21%. These experimental results demonstrate the importance of local and global features for bacterial segmentation and prove that the proposed dual-branch encoder can effectively extract these two features.

#### (2) Ablation experiments for DCA

When the DCA module was embedded in the dual-branch encoder, the segmentation performance was significantly improved. Compared to the baseline with the dual-branch encoder, the Dice for Kpn and Pae improved from 59.07% to 63.73% and from 68.89% to 69.26%, respectively, and the Dice for all categories of bacteria improved by 1.73%. The experimental results prove that the DCA module can optimize the fusion between local and global features and significantly improve the performance of the network.

#### (3) Ablation experiments for FAF

Finally, when the FAF module was applied to the baseline together with the above two modules, the segmentation performance was optimal. Specifically, the proposed network achieved 70.57%, 65.36%, and 70.49% on Dice for Aba, Kpn, and Pae, respectively, and the Dice for all categories of bacteria reached 68.73%. These ablation experiments prove that the proposed modules are effective and facilitate the precise segmentation of bacteria.

**Fig. 7:**
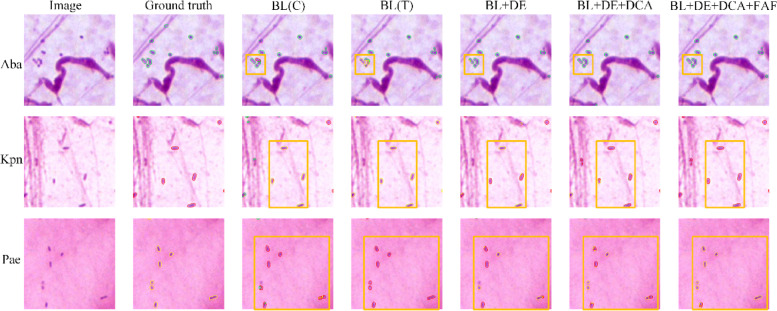
Visualized segmentation results in ablation experiments. BL(C) represents Baseline (CNN encoder), BL(T) represents Baseline (transformer encoder), and DE represents Dual-branch encoder. The region where the segmentation results are significantly improved are highlighted in orange boxes

Furthermore, to investigate the specific performance of each change compared to the baseline in the ablation experiments, we visualized several representative segmentation results, as shown in Fig. 7. It can be seen that each of the proposed modules can improve some mis-segmented regions, further demonstrating their effectiveness. In addition, we believe that the modest performance improvement of Aba is due to the fact that many Aba bacteria are not readily confused with bacteria belonging to the other two categories due to their distinctive spherical morphology. Therefore, the fine-tuned U-Net can achieve relatively good segmentation results for Aba. The proposed modules are primarily used to address the problem of misclassification of bacterial categories; as a result, they primarily enhance the segmentation accuracy for Kpn and Pae, while the performance improvement for Aba is relatively low.

### Statistical significance assessment

To investigate the statistical significance of the performance improvements of the proposed method in the comparison experiments, we performed a statistical analysis of the results for all test data using a paired *t*-test. As shown in Table 5, compared with the other SOTA methods, all the improvements for Dice and Jaccard of DB-DCAFN were statistically significant, with *p*-values less than 0.05. We also performed a statistical analysis of the results of the ablation experiments, the results of which are listed in Table 6. It can be found that, with the exception for the dual-branch encoder and baseline (transformer encoder), the *p*-values of paired *t*-tests for all ablation experiments are less than 0.05. Thus, the statistical significance of the performance improvement of the proposed DB-DCAFN was verified.

**Table 5:** Statistical analysis (*p*-value) of the proposed DB-DCAFN compared with other SOTA methods

Methods	Dice	Jaccard
DB-DCAFN-U-Net	< 2E-7	< 2E-8
DB-DCAFN-CE-Net	< 2E-4	< 1E-4
DB-DCAFN-UCTransNet	0.0014	< 2E-4
DB-DCAFN-Swin-Unet	< 1E-4	< 3E-5
DB-DCAFN-TransUNet	0.0340	0.0342

**Table 6:** Statistical analysis (*p*-value) of the results in the ablation experiment

Methods	Dice
Dual-branch encoder-Baseline1(CNN encoder)	0.0239
Dual-branch encoder-Baseline2(transformer encoder)	0.1478
Dual-branch encoder + DCA-Baseline1(CNN encoder)	< 9E-6
Dual-branch encoder + DCA- Baseline2 (transformer encoder)	0.0049
Dual-branch encoder + DCA -Dual-branch encoder	0.0122
Dual-branch encoder + DCA + FAF- Baseline1(CNN encoder)	< 7E-7
Dual-branch encoder + DCA + FAF- Baseline2(transformer encoder)	< 4E-6
Dual-branch encoder + DCA + FAF- Dual-branch encoder	< 4E-4
Dual-branch encoder + DCA + FAF- Dual-branch encoder + DCA	0.0167

## Discussion

### Effectiveness of the proposed DCA module

**Fig. 8:**
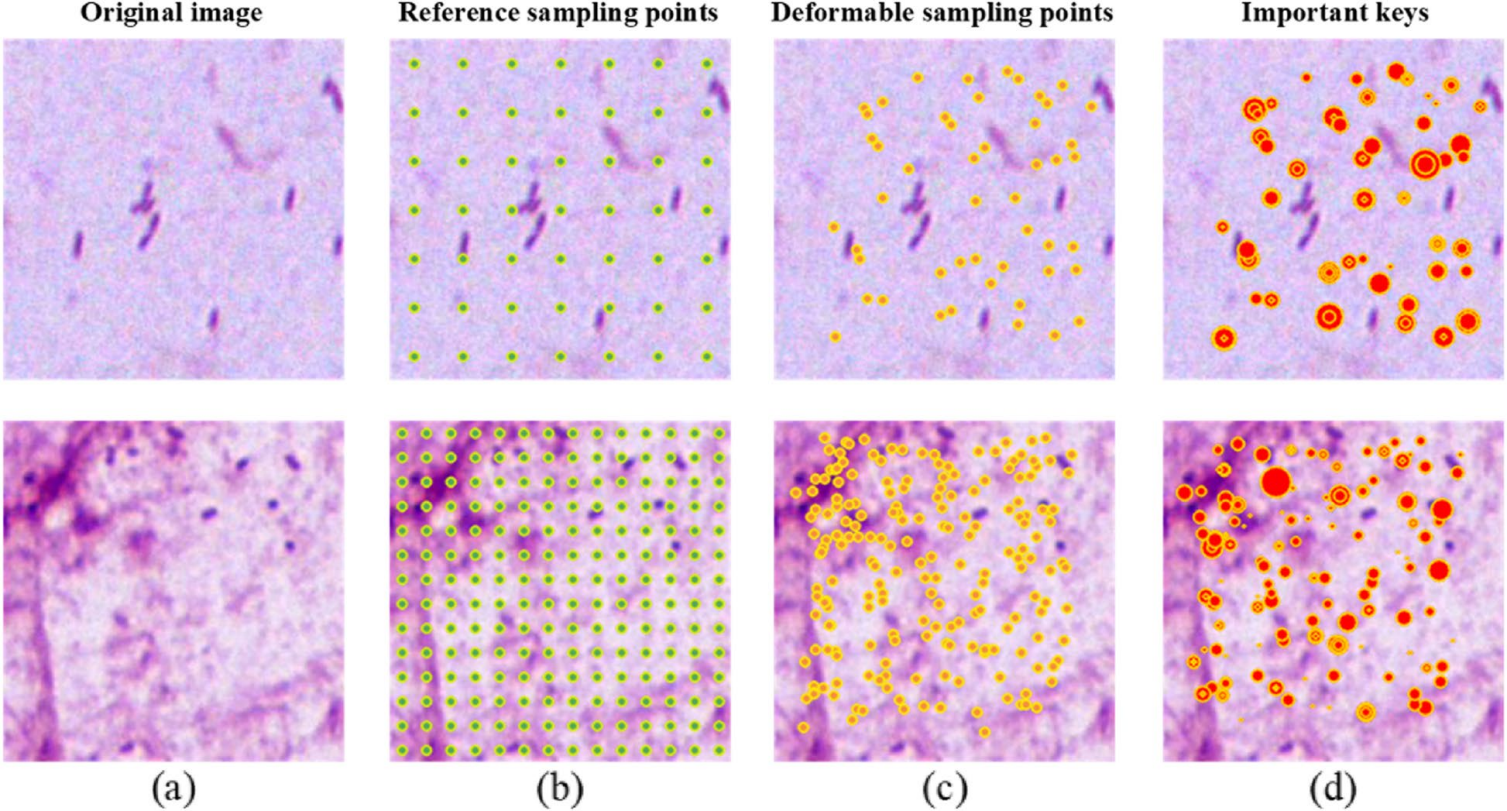
Visualizations of deformable sampling points and the most important keys in the DCA module. The green and orange circles represent the original reference sampling points and deformable sampling points, respectively. The red circle represents the most important key of each layer in multi-head cross-attention, with larger radius indicating higher key score

To further verify the specific effects of the DCA module, we visualized the process of deformable point generation and the locations of the most important keys in the DCA module. Figure 8a shows the original input image, and the reference sampling points are shown as green circles in Fig. 8b. It is worth noting that the sampling process is performed on the feature map. For better visualization, we upsampled all the sampled point coordinates to match the original image size. The deformable sampling points generated by the DCA module are indicated by orange circles in Fig. 8c. Most of the sampling points moved closer to the surroundings of the bacteria from a fixed position, indicating that the DCA module focused on the features around the bacteria when exploring more meaningful features. In addition, we visualized the most important key of each layer in multi-head cross-attention, as shown in Fig. 8d. The radius of the red circle represents the key score; a larger radius indicates a higher score. The key scores around the bacteria and complex regions are larger, which further proves that the features in the area around the bacteria and complex regions receive more attention during feature fusion. Furthermore, we investigated the different performances when applying deformable sampling to local and global features. The results are listed in Table 7, which shows that the network performance is better when offset points are applied to global features. Therefore, we applied deformable sampling to the global features of the DCA module.

**Table 7:** Results of comparison experiments about DCA module

Methods	Dice (mean ± SD, %)			
	Aba	Kpn	Pae	All
DCA (apply offset points on local features)	66.67 ± 12.79	65.43 ± 16.13	69.42 ± 14.66	67.23 ± 14.78
DCA (apply offset points on global features)	**70.57 ± 10.63**	65.36 ± 20.62	**70.49 ± 14.90**	**68.73 ± 16.26**

### Influence of applying the DCA module at different stages

The proposed DCA module can be conveniently embedded in any stage of the encoder, and the use of the DCA module at different stages may have different effects on the network. In this section, the effects of applying the DCA module at different stages of the dual-branch encoder are investigated. As shown in Table 8, the performance of the DCA module at stages 3 and 4 was better than that of the DCA module at stage 4 only. However, the performance decreased substantially when the DCA module was used in stages 2, 3, and 4. We believe that this is because the features extracted directly from the image by a single convolution layer in the patch partition are inefficient. The inefficient features not only do not bring global features but also increase the burden on the decoder, which can seriously interfere with the decoding process. Therefore, in this study, we used the DCA module in stages 3 and 4, whereas we directly fed the features extracted by the ResNet block into the decoder via the FAF modules in stages 1 and 2.

**Table 8:** Quantitative evaluation of ablation experiments on applying DCA at different stages of the encoder

Stages w/DCA module			Params (M)	Flops (G)	Dice (mean ± SD, %)	Jaccard (mean ± SD, %)
Stage 2	Stage 3	Stage 4				
		√	**17.98**	**10.89**	68.33 ± 16.09	53.69 ± 17.34
	√	√	20.21	12.31	**68.73 ± 16.26**	**54.44 ± 17.03**
√	√	√	20.78	13.76	61.17 ± 13.67	45.46 ± 14.29

√ means that the DCA module is applied to this stage

### Limitations

**Fig. 9:**
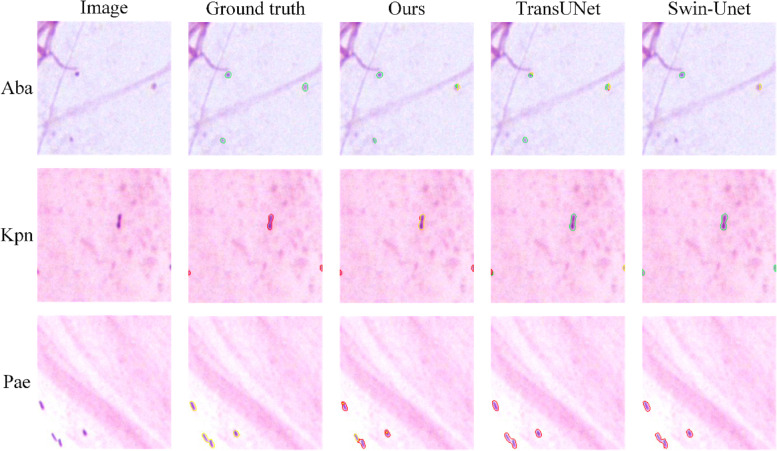
Visual comparisons of failure cases with different methods

We found that when an image patch contained a small number of bacteria, the probability of bacterial misclassification increased significantly. This is because there is less information in the image at this time, and the model can only judge the category of bacteria based on the limited features around the bacteria, while it is difficult to make use of contextual information, such as the distribution of bacteria. As shown in Fig. 9, when the number of bacteria in the field of view was limited, our method and the other SOTA methods misjudged some bacterial categories. However, the segmentation results of our method are the closest to the ground truth, and our DB-DCAFN still outperforms other competitors.

## Conclusions

In this study, we propose a novel and efficient network called DB-DCAFN to address the challenges of bacterial segmentation. In contrast to traditional CNN-based, transformer-based, or CNN-transformer cascaded encoders, our dual-branch parallel encoder consists of multiple parallel ResNet blocks and Swin Transformer blocks, which can simultaneously extract multilevel local and global context features. The DCA module can adaptively model the potential relationship between local and global features, sample sparse and meaningful global features for cross-attention, and bridge the semantic gap during feature fusion while reducing the computation cost, thus significantly boosting the model performance. In addition, our FAF module can adaptively enhance useful features during skip connections, thus further improving the segmentation precision. The experimental results on a real sputum smear dataset demonstrated that our method can efficiently segment bacteria and outperform the other five SOTA methods. Visual segmentation results showed that our method consistently performed well on ambiguous and easily misclassified bacteria. Despite achieving remarkable results, the proposed DB-DCAFN still misclassified several bacteria, as shown in Fig. 9. In future work, we will improve the network structure and reduce the number of model parameters such that it can be applied to sputum smears with a larger field of view to avoid the above problems and achieve more accurate bacterial segmentation. We will also continue to collect new categories of bacteria, explore the performance of our model on these bacteria, and evaluate its effectiveness on images with more than one category of bacteria present.

## Data Availability

The datasets used in this study are available from the corresponding author upon request.

## Abbreviations

DB-DCAFN: Dual-branch deformable cross-attention fusion network
DCA: Deformable cross-attention module
FAF: Feature assignment fusion
Aba: *Acinetobacter baumannii*
Kpn: *Klebsiella pneumonia*
Pae: *Pseudomonas aeruginosa*
ZN: Ziehl-Neelsen
FD: Fourier descriptor
CNN: Convolutional neural network
FCN: Fully convolutional network
Eco: *Escherichia* *coli*
SOTA: State-of-the-art
DAT: Deformable attention
Dice: Dice similarity coefficient
W-MSA: Window-based multi-head self-attention
SW-MSA: Shifted window-based MSA module
